# Counting Sexes and Gender: Reflections on a Timeless Tension at the Heart of Psychology

**DOI:** 10.1007/s10508-025-03312-1

**Published:** 2025-10-15

**Authors:** Lucas B. Mazur, Tom Mazur

**Affiliations:** 1 Department of Psychology, Sigmund Freud University, Columbiadamm 10, Turm 9, 12101 Berlin, Germany; 2 Institute of Pedagogy, Jagiellonian University, Krakow, Poland; 3 Departments of Psychiatry and Pediatrics, Jacobs School of Medicine and Biomedical Sciences, University at Buffalo, Buffalo, NY USA

“I am satisfied with a free wild Nature: You seem to me to cherish and pursue an Italian Garden, where all things are kept in separate compartments, and one must follow straight-ruled walks. Of course Nature gives material for those hard distinctions which you make, but they are only centers of emphasis in a flux for me; and as you treat them reality seems to me all stiffened.”

William James to Hugo Münsterberg, June 28, 1906.

Two questions were asked in the call for commentaries to stimulate thoughtful discussion and debate: “How many sexes are there?” and “How many genders are there?” These two questions appear to possess an arresting novelty. They arise from a tempest of current political, societal, ethical, medical, and moral debates, and thus also appear to possess a unique urgency. However, we will argue that what stands behind these two questions is an age-old puzzle within the study of humanity. Namely, these two questions can be understood as contemporary iterations of the debate between naturalistic psychology and interpretive psychology. While intimately interwoven with each other, these two approaches are not easy bedfellows. Both worry about being compromised by the other. To the extent that we wish for a psychological science, encroachments of interpretive approaches appear to taint the discipline, while to the extent that we assert humanity and human culture to contain a creative freedom that breaks out of causal determinism, we may feel threatened by scientific statements regarding the influence of biological or even social determinants, talk of averages, probabilities, etc. Neither approach alone can grasp the person in their entirety, nor does their simple combination present the complete picture—as if we were putting together a puzzle.

While the tension between these two positions can be confusing and frustrating, we will argue that it is precisely within this tension that we stand a better chance of appreciating the complexities of humanity. Unfortunately, most psychologists today know relatively little about the history of the field, including psychology’s close relationship with philosophy. We believe that it is crucially important for psychologists studying sex and gender to keep in mind this fundamental and irresolvable tension within the study of our psychosocial lives. A better appreciation of this tension will help psychologists recognize the enduring foundations on which the questions of today are built and thereby stand a better chance of keeping their professional calm in the face of the various storms that arise with such ostensible novelty and urgency. This informed and confident calm will then allow psychologists to productively participate—as psychologists—in the conversations and debates of the day.

## Naturalistic and Interpretive Perspectives

The naturalistic approach is an attempt to place psychology firmly and even unilaterally in the camp of the natural sciences. It is built on the assumption that “brute data” (i.e., the decontextualized and uninterpreted raw data of experiments, surveys, brain scans, etc.) are self-contained and self-explanatory and that necessary, causal relationships between such facts-in-the-world can be identified in a manner that needs little to no interpretation. This approach places a premium on various characteristics of research practices borrowed from the natural sciences, such as presumed variable isolation, causal inferences, prediction, replicability, and ahistoricity. By contrast, interpretive psychology is an attempt to describe human psychological life as embedded in wider webs of meaning, stressing the importance of irreplicable uniqueness, history, biography, cultural context, and holism. While not denying the potential usefulness of naturalistic methods, rather than seeing these methods as providing raw “facts,” interpretive approaches assert the fundamentally symbolic nature of what these methods produce (Taylor, 1985).

This basic tension in psychology has existed since the beginning of the field. Psychologists have long asserted the importance of drawing distinctions between higher-order psychological processes and the biological foundations of more basic functions; various versions of biology’s “short leash” and culture’s “long leash” (Dilthey, 1894/1977; Valsiner, 2012). This is precisely why the founders of empirical, experimental psychology, such as Wilhelm Wundt, tried to limit the scope of psychological “science” to basic functions for which consensus on conceptualization and empirical operationalization can be achieved (Danziger, 1990). The fact that these calls were and remain largely ignored can account for a considerable amount of confusion in the field, such as our perennial collective surprise when our scientific findings turn out to be considerably less scientific than we had thought (e.g., the recent “replication crisis”). The problem arises when we apply scientific methodologies and methods to concepts that are ultimately beyond their scope. While science is powerful, it is not all powerful. Most of what we are interested in within the broad field of psychology is not easily contained within a natural science framework. The fact that concepts of importance in psychology cannot be unequivocally empirically operationalized goes a long way in explaining the tremendously low predictive power of psychology relative to the natural sciences. For example, when it comes to higher-order psychological processes, quantification is of limited value. While numbers can be helpful as a symbolic system by which to facilitate communication—i.e., it can give us a ballpark depiction of symbolic phenomena—to the extent that psychometrics is believed to constitute true measurement it is bound to disappoint (Uher, 2021, 2022; Uher et al., 2025). Measuring a table is not the same thing as measuring attitudes, identities, desires, etc. “A flippant person has asked why we say, ‘As mad as a hatter.’ A more flippant person might answer that a hatter is mad because he has to measure the human head” (Chesterton, 1908/2015, p. 7).

Dilthey (1894/1977) spoke of this tension in terms of the difference between analytical psychology and descriptive psychology. Within his philosophy of social science, Max Weber spoke of it in terms of the ethics of responsibility and the ethics of conviction. It lies at the core of debates around the concept of intelligence, and those between personality psychology and differential psychology. It is one of the puzzles with which Ludwig Wittgenstein struggled. Each attempt to resolve this core bundle of tensions has been, and will continue to be, found lacking. For example, at the start of the 20th century, William Stern argued that Wilhelm Windelband’s distinction between nomothetic and ideographic perspectives and Heinrich Rickert’s objective, lawful science and subjective, values-based culture did not sufficiently recognize, let alone resolve, this fundamental tension (Lamiell, 2021). In short, while it is easy to speak of a peaceful interweaving of science and the humanities within psychology—that we recognize and use both scientific and humanities-based approaches to understanding humanity—this core bundle of tensions that challenged and even troubled psychologists, sociologists, and philosophers a century ago is very much alive today. Precisely because so few psychologists fully appreciate the history of this core tension, the field is largely left to face each new iteration thereof as if it were completely new.

## Naturalistic and Interpretive Perspectives Within the Study of Sex and Gender

“The truth is, sex is defined a lot of ways in science, but in none of them should individuals who are part of nature be ignored and excluded. That would be bad science” (Clancy et al., 2024, p. 81). While one can praise and criticize different aspects of this statement, what is important for our current discussion is that it is suggestive of the broader collective struggle to recognize and value both a scientific and interpretive understanding of humanity. It is a call to do both good science and to recognize the human “more-than-ness” that stands in contrast to the strictly scientific. The tension between the two is precisely that, a tension, because they do not exist in isolation from each other. They not only influence each other as fields of thought, each pushing and pulling the other in different directions, but they overlap even more deeply within the human phenomena they study.

Naturalistic approaches can help to assert the fundamental importance of the physical, biological world that exists irrespective of our higher-order meaning-making processes. For example, humans are a sexually dimorphic species (Strauss et al., 2023). Sex in humans is constant over time and does not change. Within this dimorphism, we find the kinds of nuanced variation one would expect from any such pattern in biological life. For example, both males and females may be born without reproductive structures or with gonadal dysgenesis, rendering them unable to produce gametes and thus infertile. People may have chromosomal patterns that are different than expected. While these conditions, known as disorders/differences of sexual development (DSDs) are very rare (Sax, 2002), they have been used to argue against the sexual binary. However, others have argued they remain within the binary because such individuals do indeed belong to one of the two sexes and that to imply otherwise can itself be ethically and scientifically problematic (Social Research Institute, 2025).

In such a view, these rare cases can be understood as the natural variation that highlights the more stable pattern(s) (Arboleda et al., 2014). A pattern does not deny variation nor is the recognition of variation a denial of the pattern. To say that humans are bipedal is not to assert that everyone has two legs or that all legs are exactly the same, while at the same time to recognize this variability does not undercut the assertion that humans are bipedal. The power that comes from asserting that humans are bipedal, have opposable thumbs and binocular vision, exhibit stereo color vision, are sexually dimorphic, etc., comes not from a denial of variations from these patterns, but rather from the more basic assertion of what constitutes pattern and variation in the first place. Science can then set about further studying and practically engaging with rule and variation alike.

There is tremendous power in both pattern and variation from the pattern, but the distinction is of crucial importance. The fact that not everyone who smokes cigarettes will develop cancer does not disprove the carcinogenic nature of cigarettes. Such variation may, in fact, be very important for better understanding the nature of the pattern. While variation is inevitable, interesting, and often even crucially informative, it does not by itself undercut the power of the pattern. It is important to highlight that—all things being equal—discussion of pattern and variation do not constitute moral judgment. What is more, a variation can be a handicap, an advantage, and/or simple a difference in various contexts. That having been said, we can certainly challenge cultural associations or stereotypes related to such patterns and variations.

The same is true in the other direction. No matter how hard we may try, the scientific study of humanity cannot fully free itself from the importance of higher-order, meaning-making processes. For example, when the biological sex of a newborn is announced, this is both an expression of biological knowledge and a symbolic act of considerable psychosocial consequence (Money & Ehrhardt, 1972). The concepts “man” and “woman” are expressions of semiotic mediation (Valsiner, 2007), which is to say that even though they speak to “things-in-the-world,” they necessarily do so through the filter that is higher-order processes of meaning-making. What is more, an interpretive psychology asserts the fundamental importance of the person behind the statistic, the classification, and beyond the reach of biological determinism. Here is how William Stern wrote about this broader issue in 1918:

Despite all commonalities through which persons are like other instances of humanity, representatives of a race, members of a gender, despite all broader and narrower lawful regularities that are at play in all personal happenings, there ever remains an ultimate reality, according to which every person stands as a world unto himself before every other person. (translated in Lamiell, 2021, p. 50)

It is precisely here that the human defies classification and measurement not by means of variation, but by being immeasurable, innumerable, something more-than. The person cannot be fully contained within the scope of the scientific method(s). Naturalistically minded psychologists tend to underappreciate, or even completely overlook, the fact that most psychological phenomena do not meet the basic criteria of scientific research (e.g., replicability, experimental control, causality, and prediction) (Bruner, 1990; Mazur, 2021; Valsiner, 2012). Following our collective surprise at the recent “replication crisis,” most mainstream attempts to address this crisis have overlooked the fundamental differences between naturalistic and interpretive approaches, opting rather to further “tighten the screws” of naturalistic approaches (e.g., by requiring the preregistration of research plans and hypotheses, the publication of raw datasets). These approaches will not pull psychology more to the side of science and they will not silence the interpretive voices that remind us that human meaning-making processes cannot be enclosed within naturalistic methodologies. On the other side, interpretively minded psychologists look at the various ways humans break free from science’s “short leash,” and often misconstrue the nature of, and downplay the importance of, basic biological determinism. However appealing, such positions will not definitively free psychology from the reach of the natural sciences. What is important for our current considerations is that these debates within research on sex and gender are at their core contemporary iterations of the more basic tension between naturalistic and interpretive approaches. This tension will never be resolved precisely because the person cannot be contained within either approach nor within a combination of the two. This basic epistemological humility can go a long way, especially in the face of the various storms that accompany each new version of this tension, be they political, cultural, societal, moral, medical, ethical, etc.

## Conclusion: Counting Sexes and Gender

Research within the field of science studies has long convincingly shown that even the most clearly naturalistic of sciences exhibit the presence of human meaning-making processes (Hess, 1997) and, thus, that interpretive approaches are important for understanding the workings of those fields. On the other hand, advances in the natural sciences have been so undeniably powerful as to render no field or school of thought fully beyond their reach, even interpretive approaches. Put more directly, in order to more fully understand humanity, not only are both naturalistic and interpretive approaches needed, but the core bidirectional tension between them is unavoidable.

This point is nicely illustrated by the fact that we use the same terms for subcategories within sex and gender (i.e., male and female). While it can be theoretically and practically helpful to distinguish between sex and gender (e.g., during data collection, Social Research Institute, 2025), that the concepts male and female refer to both a scientific, biological understanding of who we are and an interpretive, humanistic understanding, wonderfully attests to how these two approaches are fundamentally interwoven. In other words, the confusing mixture of biology and higher-order meaning-make processes found with such concepts as male and female is not simply a matter of semantics, as if this confusion could be avoided by simply using different terms for the subcategories of sex and gender. As biological beings, humans are sexually dimorphic, with all the variation this entails. At the same time, as beings whose psychosocial, cultural meaning-making processes are a step removed from biological determinism, what our bodies mean to us and to others, how we choose to give psychological or social expression to those bodies, is necessarily beyond the reach of naturalistic science. Biological sex affects our physical, psychosocial, and cultural lives, but once we reach these higher-order processes we are no longer in the realm of (solely) the natural sciences. There is no one way to live on the basis of one’s body and, thus, there is no one way to psychologically experience one’s fitting or not fitting the expectations that may be individually or socially associated with our bodies. This is true for people whose bodies do not fit particularly well within the binary, but it is also true for people whose bodies do fit relatively well therein. It is precisely here that the human defies classification and measurement not by means of variation, but by being something immeasurable, innumerable, something more-than. It is not so much that there are too many genders to count, as that it simply makes no sense to count in the first place. In other words, in as far as gender is not coterminous with sex and refers rather to our higher-order psychosocial engagement with biological sex, this is not a matter best served by rigid quantification, enumeration, or classification. Reflecting on this tension at the start of 1900s, Weber (1906/1949) wrote that the “social sciences, which are strictly empirical sciences, are the least fitted to presume to save the individual the difficulty of making a choice, and they should therefore not create the impression that they can do so” (p. 19).

While this may disappoint naturalistically minded utopists and anti-science humanists alike, the majority of us should rejoice. Rather than despairing at our inability to resolve this tension, we should follow the advice of the social psychologist Gustaw Ichheiser, who argued that psychologists “should cheerfully accept the fact that what they are doing belongs to the twilight zone between science and literature” (cited in Rudmin et al., 1987). When it comes to matters of sex and gender, there are times when it is helpful to count and classify, and times when it is not. The decision of which approach is more fitting depends on the particularities of the given situation before us and the nature of the questions we are asking. However, it can be tremendously helpful to remain mindful of the fact that regardless of the approach we take, the fundamental tension of which we have been writing will remain. This can help to remind us that the human person is in the foreground, ahead of the methodological lens we are using, whichever lens that may be. That we can never fully extricate ourselves as a field from this balancing act between science and the humanities attests not to the weakness of a compromised psychology, but to the strength and ongoing relevance of a psychology that is willing to reflect, confer, and epistemologically compromise in the face of human complexity.

## Data Availability

Not applicable.

## References

[CR1] Arboleda, V. A., Sandberg, D. E., & Vilain, E. (2014). DSDs: Genetics, underlying pathologies and psychosexual differentiation. *Nature Reviews Endocrinology,**10*(10), 603–615. 25091731 10.1038/nrendo.2014.130 PMC4441533

[CR2] Bruner, J. (1990). *Acts of meaning*. Harvard University Press.

[CR3] Chesterton, G. K. (1908/2015). *Orthodoxy*. Snowball Classics.

[CR4] Clancy, K., Fuentes, A., VanSickle, C., & Clune-Taylor, C. (2024). Biology is not binary. How scientists define sex has evolved over the centuries, and the concept is still incredibly difficult to pin down. *American Scientist,**112*(2), 78–81. 10.1511/2024.112.2.78

[CR5] Danziger, K. (1990). *Constructing the subject: Historical origins of psychological research*. Cambridge University Press.

[CR6] Dilthey, W. (1894/1977). *Descriptive psychology and historical understanding* (Trans. R. M. Zaner & K. L. Heiges). Martinus Nijhoff.

[CR7] Hess, D. J. (1997). *Science studies: An advanced introduction*. NYU Press.

[CR8] Lamiell, J. (2021). *Uncovering critical personalism: Readings from William Stern’s contributions to scientific psychology*. Pelgrave.

[CR21] Mazur, L. B. (2021). The epistemological imperialism of science. Reinvigorating early critiques of scientism. *Frontiers in Psychology*, *11*. 10.3389/fpsyg.2020.609823 10.3389/fpsyg.2020.609823 PMC7817851 33488476

[CR9] Money, J., & Ehrhardt, A. A. (1972). *Man & woman, boy & girl: The differentiation and dimorphism of gender identity from conception to maturity*. Johns Hopkins University Press.

[CR10] Rudmin, F., Trimpop, R. M., Kryl, I., & Boski, P. (1987). Gustav Ichhieser in the history of social psychology: An early phenomenology of social attribution. *British Journal of Social Psychology,**26*, 165–180.

[CR11] Sax, L. (2002). How common is intersex? A response to Anne Fausto-Sterling. *Journal of Sex Research,**39*(3), 174–178. 10.1080/00224490209552139 12476264 10.1080/00224490209552139

[CR12] Social Research Institute. (2025). *Review of data, statistics and research on sex and gender*. Available online: https://www.gov.uk/government/publications/independent-review-of-data-statistics-and-research-on-sex-and-gender

[CR13] Strauss, J. F., Barbieri, R. L., Dokras, A., Williams, C. J., & Williams, S. Z. (2023). *Yen & Jaffe’s reproductive endocrinology* (9th ed.). Elsevier.

[CR14] Taylor, C. (1985). Peaceful coexistence in psychology. In C. Tayor (Ed.), *Human agency and language* (pp. 117–138). Cambridge University Press.

[CR15] Uher, J. (2021). Psychometrics is not measurement. Unraveling a fundamental misconception in quantitative psychology and the complex network of its underlying fallacies. *Journal of Theoretical and Philosophical Psychology,**41*, 58–84. 10.1037/teo0000176

[CR16] Uher, J. (2022). Rating scales institutionalise a network of logical errors and conceptual problems in research practices: A rigorous analysis showing ways to tackle psychology’s crises. *Frontiers in Psychology,**13*, 1009893. 10.3389/fpsyg.2022.1009893 36643697 10.3389/fpsyg.2022.1009893 PMC9833395

[CR20] Uher, J., Arnulf, J. K., Barrett, P. T., Heene, M., Heine, J.-H., Martin, J., Mazur, L. B., McGann, M., Mislevy, R. J., Speelman, C., Toomela, A, & Weber, R. (2025). Psychology’s questionable research fundamentals (QRFs): Key problems in quantitative psychology and psychological measurement beyond questionable research practices (QRPs). *Frontiers in Psychology*, *16*,1553028. 10.3389/fpsyg.2025.1553028 10.3389/fpsyg.2025.1553028 PMC12414940 40927341

[CR17] Valsiner, J. (2007). *Culture in minds and societies*. Sage.

[CR18] Valsiner, J. (2012). *A guided science: History of psychology in the mirror of its making*. Transaction.

[CR19] Weber, M. (1906/1949). *The methodology of the social sciences*. The Free Press.

